# Young Adults’ Experience of Loneliness in London’s Most Deprived Areas

**DOI:** 10.3389/fpsyg.2021.660791

**Published:** 2021-05-24

**Authors:** Sam Fardghassemi, Helene Joffe

**Affiliations:** Division of Psychology and Language Sciences, University College London, London, United Kingdom

**Keywords:** loneliness, young adults, social media, coping, deprived areas

## Abstract

Young adults (16–24 years old) are currently the loneliest group in Western countries. In particular, young adults of lower socio-economic status (SES) living in the most deprived areas are loneliest in the United Kingdom. This mixed-methods study explored the experience of loneliness among this under-explored demographic in London. Using a novel free association technique, the experience of loneliness was found to be characterized by: a sense of isolation, negative emotions and thoughts, coping and a positive orientation to aloneness. An exploration of these themes revealed that: one can feel isolated or excluded even when surrounded by people; the experience of loneliness is accompanied by a set of interrelated feelings and thoughts like rumination; and technological and/or non-technological outlets can be used to cope. Social media play both a positive and negative role in loneliness, and loneliness is not always experienced negatively. The quantitative data indicated that this sample was lonely. By providing insight into young adults’ loneliness, the findings indicate what types of interventions are likely to diminish it.

## Introduction

Loneliness has become a major public health pandemic. Although most scientific and media interest in loneliness has historically concerned older adults, there is new evidence that young adults, specifically those between 16 and 24 years old, are particularly vulnerable to loneliness. This was the case prior to the exacerbation of loneliness that the Covid-19 pandemic has created (Office for National Statistics, 2021). Prior to the Covid-19 pandemic, the largest survey on loneliness globally showed that 40% of young adults between the ages of 16 and 24 said they felt lonely often or very often, while 29% of older adults aged 65–74 and 27% of those aged 75+ said this (BBC Loneliness Experiment, 2018). Corroborating this, surveys from the United Kingdom and other developed countries including the United States and Japan have found that young adults are most likely to experience loneliness (Cigna, 2018, 2020; DiJulio et al., 2018; Office for National Statistics, 2018a). In the United Kingdom, nearly a quarter (24%) of young adults (18–24 years old) say they often experience loneliness and 7% report they are lonely all of the time (YouGov, 2019).

Loneliness among young adults is associated with a number of physical and mental health problems, including a weaker immune system, poorer sleep, greater psychological stress, increases in depressive symptoms and anxiety (Cacioppo et al., 2002, 2006; Pressman et al., 2005; Wei et al., 2005; Griffin, 2010; Vanhalst et al., 2012). In the United Kingdom, specifically, lonely vs. non-lonely people aged 16–80+ are seven times more likely to have low life satisfaction, 10 times more likely to report low feelings of worth, three times more likely to report feeling unhappy, and twice as likely to report feeling anxious (Office for National Statistics, 2015). Lonelier young adults have also been found to be more likely than their non-lonely counterparts to engage in risky physical health behaviors and to use negative coping mechanisms such as withdrawing or obsessing about problems to deal with stress (Matthews et al., 2019). Collectively, these data indicate that young adults are currently at the greatest risk of suffering from loneliness and its consequences, which makes it imperative to examine the experience of loneliness among them.

The present study was designed to explore the specific experience of loneliness in those identified by surveys as falling into the loneliest demographic: young adults of lower socio-economic status (SES), in employment, renting and living in the most deprived areas (Office for National Statistics, 2018b) of London. This study provides a systematic, in-depth qualitative analysis of the experience of loneliness and experience of coping with it in those most likely to be lonely. The interviews are accompanied by questionnaires that provide quantitative data on young adults’ degree of loneliness and engagement with social media.

The multidimensional theory of loneliness (Weiss, 1973) postulates that there are different types of loneliness: “social” and “emotional.” While social loneliness is experienced when one feels a lack of a social network (i.e., friends, colleagues, and people in one’s neighborhood), emotional loneliness is felt when there is an absence of intimate relationships or close attachments (i.e., a partner or best friend). Thus, one can experience emotional loneliness even if one has a large social network. Similarly, one can feel socially lonely despite having intimate relationships. Weiss considers the emotional aspect to be a more intensely painful form of loneliness because one feels one lacks relationships that are close and deep. Weiss’ theory of multidimensional loneliness is well supported empirically (e.g., Hyland et al., 2019). More specifically, one study examining developmental trends regarding loneliness found that while emotional loneliness intensified across adolescence and young adulthood, social loneliness lessened (Von Soest et al., 2020). With the increasing use of social media in contemporary culture and focus upon growing one’s virtual friendship network in the form of numbers of “friends” or “followers,” young adults may be at risk of lacking depth and meaning in the quality of their online friendships and ultimately feeling emotionally lonely.

The evolutionary model (Cacioppo and Cacioppo, 2018) offers another perspective with which to explore loneliness. It proposes that the experience of loneliness signals that important social relationships are of low quality, under threat or absent (Cacioppo and Hawkley, 2009), and encourages people to fix the deficient connections. When this happens, loneliness diminishes, which is rewarding in itself and people start to benefit from their social interactions with others again. This regulatory process of rebuilding one’s social relationships when they are experienced as deficient, has aided human evolution by contributing to increased chances of survival and reproduction (Goossens, 2018). Young adulthood is bound to a number of predictable transitory phases connected to household, relationships, employment, and education, which call for social reorientation and cause loneliness. Moreover, since many youths currently spend a large portion of their social lives on social media (Smith and Anderson, 2018); this amplifies the likelihood of being hypervigilant to social cues, which can signal threat to their sense of self or how they are perceived by others.

Although being alone is often portrayed as an undesirable state, not all aloneness is actually detrimental (Galanaki, 2013). Solitude offers time for reflection, development, self-exploration, creative activity, and an escape from social life that can recharge and renew the self (Storr, 1988; Csikszentmihalyi, 1996; Larson, 1997; Goossens, 2014; Korpela and Staats, 2014). Adolescents who spend at least some portion of their time alone appear to be better adjusted, perhaps because they create a space for self-nurturant thoughts and the development of their identities (Larson, 1990). However, spending too much alone time alone can signal depression (American Psychiatric Association, 1987).

Examining individual differences that make certain people more susceptible to loneliness may aid understanding of the growing evidence for the direct links between loneliness and low wellbeing. Intrapersonal factors such as low self-worth and personality traits including introversion and emotional instability are associated with prolonged loneliness across childhood, adolescence, and adulthood (Dykstra et al., 2005; Qualter et al., 2013; Vanhalst et al., 2013; Newall et al., 2014).

There may also be demographic factors associated with loneliness in young adults. In a meta-analysis of nearly 400,000 individuals across all age groups, there was no strong evidence for gender differences in self-reported loneliness (Maes et al., 2019). A more recent study, which used data from the large, global BBC Loneliness Experiment, however, showed that loneliness was greater in men than women, particularly younger men living in individualistic cultures (Barreto et al., 2021). Furthermore, the relationship between being ethnically different from the mainstream culture and loneliness has been explored and is strongest among young and early middle-aged adults (19–34 and 35–49 years old) compared to other age groups (Franssen et al., 2020). In another study, young British adults (16–24 years old) who identified as black or an ethnic minority were more likely to report higher levels of loneliness than those who did not (Office for National Statistics, 2018a).

In addition to personality, gender, and ethnicity, an exploratory study of the experience of loneliness among British young people (16–24 years old) and children (10–15 years old; Office for National Statistics, 2018a) found that such experiences arose in relation to disability, being bullied, having mental health challenges, exam pressure, loss of significant relationships, work commitments, cost of participation in social activities, distance from friends, living in a single-person household, and transitions linked to education and work.

Young people spend a significant portion of their social lives on social media, and this may be linked to their loneliness (Smith and Anderson, 2018). The evidence concerning the association between the two is equivocal. In one experimental study, university students were randomly assigned to either limit social media use, or to use social media as usual (Hunt et al., 2018). The limited use group showed a significant reduction in loneliness and depression over 3 weeks compared to the control group. The finding suggests that limiting social media use to approximately 30 min per day may lead to significant improvement in wellbeing. Conversely, in another experimental study, one group of young people were instructed to post more than they usually do on Facebook, while those in the control condition were not instructed to do anything (Deters and Mehl, 2013). The study found that an increase in posting on Facebook reduced feelings of loneliness over the course of the week regardless of whether status updates received a response or not. Loneliness did not change among those in the control condition. The researchers concluded that the decrease in loneliness was due to the participants who posted more feeling more connected to their friends on a daily basis. This suggests that the very act of sharing on social media can be a way of feeling that one is connecting with one’s social network.

Such inconsistent findings are also evident in correlational studies. For example, in one study, adolescents high in social media use and low in face-to-face social interaction reported high levels of loneliness (Twenge et al., 2019). However, in another, social media use among university students was negatively associated with loneliness (Yavich et al., 2019).

The relationship between social media and the experience of loneliness may depend on how it is used. For example, a meta-analysis showed that while active social media use such as to update one’s status or share photos was linked with greater wellbeing, passive consumption including browsing or observing others but not interacting with them was associated with poorer wellbeing (Liu et al., 2019). Other researchers caution that the impact of social media on loneliness is not “black and white” and that even within active use different levels of use have differential consequences. For instance, adolescents who heavily engaged with social media by constantly updating their status and sharing photos increased their sense of loneliness whereas those with low to moderate levels of broadcasting and posts reduced it (Wang et al., 2018). This suggests that perhaps sharing too much information on social media may increase one’s dependence on the platforms for feedback and approval from others and decrease the opportunity for the development of intimate ties and a rich network of social relationships. On the other hand, low to moderately active users may have used social media for purposes such as nurturing existing relationships thereby explaining the concomitant decrease in loneliness.

The literature also provides some insight into how young people cope with loneliness. An early study found that adolescents in rural United States used coping mechanisms, such as listening to music, watching TV, exercising, playing with pets, using positive affirmations, and calling relatives while a smaller number ascribed to strategies, such as smoking, drinking, and using drugs (Woodward and Frank, 1988). In a more recent study, coping mechanisms of British and international university students living in the United Kingdom who had moved away from home to study and self-identified as experiencing loneliness were examined qualitatively (Vasileiou et al., 2019). A variety of strategies were found, such as: seeking support from family and friends offline and online, actively trying to make friends, comforting oneself that the difficult feelings would pass, reassuring oneself that one has close relationships, rumination, “killing time” (through using social media and the internet), studying, sleeping, and engaging in a diverse range of constructive and pleasurable activities as a distraction.

Furthermore, another study investigated the similarities in the way adolescents cope with loneliness both online and offline given the major internet use by adolescents from the 1990’s onwards. Seepersad (2004) found that youth who avoid dealing with their loneliness offline also avoid dealing with it online. Specifically, those who coped using “passive avoidant” coping (e.g., making little conscious effort to actively reduce loneliness) used the internet for entertainment purposes, such as playing video games, going on chatrooms, and surfing. However, adolescents who dealt with their sense of loneliness using an “approach” coping mechanism (e.g., expressing one’s emotions or trying to resolve the problem by communicating with others) considered communication to be an important use of the internet. Thus, online and offline coping behaviors are related.

It is important to note that while there is a large body of research on loneliness among young people (i.e., 10–19 years old); little is known about loneliness in young adults (i.e., 18–24 years old). Given that currently young adults are the loneliest group within the United Kingdom and other Western countries, even prior to the Covid-19 pandemic (e.g., Cigna, 2018; Office for National Statistics, 2018a; YouGov, 2019; and intensified during the pandemic, i.e., see Office for National Statistics, 2021), the aim of the current study is to examine the subjective experience of loneliness among this demographic. Furthermore, the focus of this research is on young adults in the United Kingdom’s capital, London, because it has been ranked as the loneliest city within the United Kingdom (ComRes, 2013).

The study is couched in a phenomenological epistemological framework (Husserl, 1900/1901, 2012). As an approach that seeks to understand the essence of a phenomenon by exploring it from the perspective of those who have experienced it, this study focuses on young adults’ lived experience of loneliness. The goal of the theory is to describe the meaning of experience both in terms of what has been experienced and how it was experienced. Pre-existing assumptions are avoided so that new understandings emerge to inform and re-orientate the field. The following research questions will be addressed:

- What is the experience of loneliness among young adults from London’s most deprived boroughs?
- Do social media impact young adults’ loneliness?
- How do these young adults cope with their experience of loneliness?

## Materials and Methods

### Participants

Forty-eight participants were recruited between May and August 2019. A recruitment agency was employed to access the quota sample required. The sample was selected based on the Office for National Statistics (2018b) correlates of loneliness: young adults, lower SES (according to the census categorization thereof) in employment (full‐ or part-time), and renting and living in the four most deprived areas of London: Newham (*n* = 16), Hackney (*n* = 16), Tower Hamlets (*n* = 16), and Barking and Dagenham (*n* = 16; English Indices of Deprivation, 2015). Participants were British born (23 males, 24 females, and one “other”), aged 18–24 (*M* = 21.23, *SD* = 2.43). Further demographic characteristics are shown in Table 1.

**Table 1:** Young adults’ (18–24 years old) demographics (in numbers and percentages)^1^.

Demographic categories	Male	Female	Other	Total
**Boroughs**				
Newham	6	5	1	12
Hackney	5	7	-	12
Tower hamlets	7	5	-	12
Barking and Dagenham	5	7	-	12
**Race**				
White	6	9	-	15 (31.25%)
Black, Asian, and Minority Ethic (BAME)	17	15	1	33 (68.75%)
**Religion**				
Christian	10	6	1	17 (37.5%)
Muslim	9	5	-	14 (29.17%)
No religion	5	7	-	12 (25%)
Other	3	0	-	3 (6.25%)
Prefer not to say	1	1	-	2 (4.17%)
**Total**	23	24	1	48

1 The questionnaire about religion presented participants with a list of options including: “Jewish,” “Buddhist,” “Hindu,”, “Sikh,” “Christian,” “Muslim,” “No religion,” “I’d rather not say,” and “Other (please specify)…”. In this table, we only included the religions or options that participants from the present study were affiliated with.

### Procedure

Interviews were arranged by the recruitment agency and participants were paid a small cash incentive. Before completing the interview, participants were provided with an information sheet that outlined the nature of the study in general terms: a research project exploring young adults’ social lives in the city. Before the study began, they were kept blind to the study’s objectives. The free association task and interviews were conducted once participants had signed a written consent form agreeing to be interviewed and audiotaped. After the interview, participants completed self-report questionnaires that ascertained loneliness, social media, and demographic data and were fully debriefed. They were also given a list of professional services in case of need for further support if the interview had elicited unwanted feelings. Finally, ethical permission was obtained from UCL’s Research Ethics Committee prior to the study (CEHP/2013/500).

### Free Association Task

**Figure 1:**
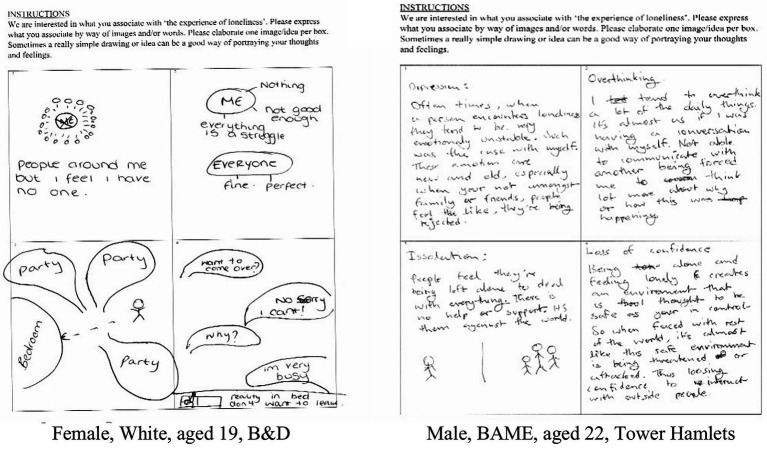
Examples of filled-in free association grids.

Free associations uncover people’s thoughts and feelings about a given issue without input from researchers concerning the range of possible responses. Thus, they provide a more naturalistic inroad into people’s conceptualizations than most methods. A free associative technique, termed the Grid Elaboration Method (GEM), gives people the opportunity to produce their own images and ideas on the assumption that this reveals conceptualizations that underpin surface level accounts (Joffe and Elsey, 2014). Based on the GEM, participants were presented with a piece of paper that contained a grid of four empty boxes and asked to express what they associated with “the experience of loneliness” by way of images and/or words. They were further instructed to elaborate one image/idea per box.^1^ See Figure 1, for examples of the free association grid output from the current study. The prompt for the free association (i.e., “connectedness with other people,” “the experience of loneliness”), was piloted with two participants from Hackney and Tower Hamlets between May and June, 2019. The pilot showed that the phrase “the experience of loneliness” was more effective. After completion of each free association task, participants were asked to elaborate on the content of each box, in turn, in an interview. This started with “can we talk about what you have put in box 1, please?” Prompts such as “can you tell me more about that?” were used to ensure thoughts and feelings about the experience of loneliness were fully explored and emerged naturalistically without injection of content *via* researcher questioning. The interviews lasted for an average of 60 min.

### Measures

#### Loneliness

Participants completed the 20-item Revised University of California, Los Angeles (UCLA) Loneliness Scale (Russell et al., 1980; *α* = 0.94). It contained 10 items with reversed scores and participants rated each item on a scale from 1 (“Never”) to 4 (“Often”). A sample item includes “I feel in tune with the people around me.” The Scale’s scoring format included the sum of the mean of all 20 items. The total score on the scale should range from 20 to 80 with higher scores indicating higher loneliness (Russell et al., 1980). The most commonly used categorization is the following: 20–34 denotes a low degree of loneliness, 35–49 denotes a moderate degree of loneliness, 50–64 denotes a moderately high degree of loneliness, and 65–80 denotes a high degree of loneliness (Russell et al., 1980).

#### Social Media

The Media and Technology Usage and Attitudes Scale (MTUAS; Rosen et al., 2013) was used to assess social media use. The scale includes 15 subscales with 60 items, however, only General Social Media Usage (nine items), Online Friendships (two items), and Facebook Friendships (two items) were used for the purpose of this study because they have a particular relevance for social media use. The nine-item General Social Media Usage subscale was assessed through a 10-point Likert scale ranging from 1 (“Never”) to 10 (“All of my waking life”). A sample item includes “Click ‘Like’ to a posting, photo, etc.” The Online Friendships and Facebook Friendships subscales were aggregated, and Facebook Friendships were modified to represent online friendships as a whole. The two combined subscales were measured with scores ranging from 1 (“0”) to 9 (“751 or more”). Higher scores meant greater number of friends. A sample item is “How many people do you regularly interact with online that you have never met in person?” The Cronbach’s alpha coefficients were 0.85 for the overall scale and 0.97, 0.83, and 0.96, for the three subscales used in this study, respectively.

Finally, participants were asked to list their top three social media channels based on frequency of use. This measure was an added question from the current researchers in order to determine what social media platforms, in particular, the participants were referring to in their responses to the above scales.

### Data Analysis

Two types of data were obtained: the interview dialogue (qualitative) and questionnaires (quantitative).

Interviews in which participants elaborated their own grids were thematically analyzed (Joffe and Yardley, 2004; Joffe, 2012). Transcripts were read thoroughly, and the key ideas contained in them noted and categorized. This allowed codes to develop naturalistically from what was observed. These codes were then grouped into sets in a coding frame. This ensured that a more complex picture of people’s thoughts was accessed, beyond the condensed, initial free associations. A reliability check of the coding manual was then performed *via* Atlas.ti 8: A second coder was trained to double-code over 10% of the data and inter-coder agreement analysis revealed an average Krippendorff’s Cu-Alpha of 0.642 across all the codes. This indicates substantial reliability (Landis and Koch, 1977). There is a current debate as to whether thematic analyses should be reliability checked (Braun and Clarke, 2020; O’Connor and Joffe, 2020); it was conducted to give the analysis a systematic underpinning. The full data set was then analyzed using Atlas.ti 8.

The quantitative data gathered from the questionnaires were entered into SPSS (IBM SPSS, 2017). These data included descriptive statistics, Pearson correlations, and a graph/bar chart.

## Results

### Results of the Interview-Based Analysis

#### Interview Themes

The following sections outline the most prevalent themes found in young adults’ elaborations of their own free associations concerning the experience of loneliness (see Table 2). Within each theme, responses were compared across gender and ethnicity.^2^ There were four key themes that emerged regarding the subjective experience of loneliness among young adults. Each theme is discussed in turn in the following sections. A symbol used commonly by participants to metaphorically capture the theme is presented at the end of each theme.

**Table 2:** The experience of loneliness among young adults in London’s most deprived areas: theme summary.

**1. Isolation (81%)^1^**
a) Feeling isolated, excluded, or left out
b) Sense of loneliness despite being surrounded by people
c) Social media and exclusion
**2. Negative feelings and thoughts (81%)**
a) Sadness and depression
b) Overthinking, being bothered by one’s thoughts, or mental rumination
c) Fear of being judged, excluded, rejected, isolated, or left out
d) Worry and anxiety
e) Low self-worth, self-esteem, or self-confidence
f) Emptiness
**3. Coping (54%)**
a) Coping mechanisms using technology
b) Coping mechanisms not using technology
**4. Positive orientation to aloneness (56%)**
a) Positive orientation to aloneness in general
b) Time and space for reflection
c) The positive experience of social media

1 This is the proportion of the sample who mentioned the theme.

### Theme 1: Isolation

#### a) Feeling Isolated, Excluded, or Left Out

The vast majority of the sample associated the experience of loneliness with a sense of isolation: in particular, being physically and emotionally isolated, excluded, or being by oneself. A significant number of the participants expressed physical and emotional isolation by way of symbolically depicting an individual on their own, feeling sad while a group of people next to them was feeling happy and having fun. There were mentions of the lonely individual being excluded, an outcast or an outsider who wanted to join the group or felt jealous about everyone in the group being together. For example:

*“Yep, so it’s um, one person with a sad face and then groups of people, couples of people with happy faces and so, when you are lonely, you are on your own and you are sad and everyone else around you is all together and they are happy. So, it’s sort of like, ex-, like the loneliness relating with sadness, I may be jealous here because everyone else is happy.”* [Female, 19, White, Newham]

The feeling of being detached was talked about in connection to both physical and emotional exclusion. One could become disconnected intentionally or detach oneself from others. Both left one feeling that there was no one there for one or who understood one.

School environments often left people feeling unwanted, excluded, or alone. People also mentioned purposefully isolating themselves if they felt depressed and did not want to burden others with their problems. Furthermore, transitionary periods such as moving from school to university or from university to work were identified as emotionally isolating because the environment and responsibilities differed dramatically. Similarly, the pressure of work was considered to be an isolating experience because it left little time for social contact.

#### b) Sense of Loneliness Despite Being Surrounded by People

A subjective sense of loneliness despite having close relationships was another factor strongly associated with the experience of loneliness. Many of the participants talked about feeling lonely even when “in a room full of people” including family and friends. Salient within this theme was not being understood, not being able to express oneself and one’s feelings, being different (e.g., sexual orientation, ethnicity, different body size, and interests) and experiencing a sense of emptiness:

*“Yea, um, being misunderstood was the first thing that I thought I associate with loneliness. I think, um, sometimes you could have, someone could be, maybe surrounded, you could still have people around you, if they do not quite understand you, you could still feel quite lonely.”*[Male, 24, BAME, Newham]

#### c) Social Media and Exclusion

The sense of isolation was also linked by many to social media. Seeing photos on social media of an event, a get-together, or holidays to which one was not invited, included or involved, contributed to the experience of loneliness. Young adults compared their experience with that of their friends, and this left them with a sense of being trapped at home alone while friends were out having fun together. Even seeing random people at a party, a special outing, or holiday resort on social media contributed to their experience of loneliness. Lack of money and physical distance from friends were also specifically mentioned as a cause of being stuck at home and unable to join friends out. The experience of seeing social media images of friends or other people seemingly having fun without one brought about feelings of sadness, jealousy, disappointment, exclusion, and questioning of one’s worth:

*“…when you see pictures that you are not involved in and it just generally seems fun, that’s when you start feeling alone because you are not… with them, you are not going to be in the background smiling or anything, it’s more you sitting down on your bed or something and you are looking, tapping, seeing that loads of photos… you cannot, cannot really escape these kinda um photos and these situations, where you are gonna bound to be and where you are not involved in.”* [Male, 18, BAME, Hackney]

Isolation was symbolized as a sense of being trapped in a perspective that is different to that of other people:

*“…you are the lion in the cage and you have got a crowd of people around you. You’re fighting to get out, but everyone’s like “oh! Entertainment in the cage.,” like they do not understand what your fight is. They just see it as, it’s like two views of the same thing. The lion is trying to get out and in the lion’s mind he is trapped. This is not a good place. It’s not fun. He does not understand the lights and the crowds and everything else that’s going on around him, it is danger. But to everyone else from their perspective is entertainment…”* [Female, 24, BAME Hackney]

### Theme 2: “Negative Feelings and Thoughts”

A large majority of the sample associated a range of negative feelings and thoughts with loneliness. The most common of these were sadness and depression, overthinking and fear, and the less prevalent included worry and anxiety, low self-worth, and emptiness. Most of the participants did not like being on their own because their experience of being alone was accompanied by these unwelcome emotional and mental states. Being at home or in one’s bedroom were the two places where young adults generally engaged in these negative states.

#### a) Sadness and Depression

Many participants associated sadness and depression with the experience of loneliness.

Beyond explicit mention of these feelings, they were alluded to by talk of “wanting to stay in bed,” “crying often,” “not interested in speaking to anyone,” “what is the point of living?.” There were also references to the feeling that one does not have anyone, especially when one wants to go out, or one is unable to express oneself:

*“…I feel like I associate being upset with sometimes feeling a bit lonely. I feel like being upset would sometimes leave you feeling a bit lonely. Because sometimes when you are feeling upset to yourself about something and you just thought of holding it in and keeping it in, that can make you feel a bit lonely…”* [Female, 22, BAME, Tower Hamlets]

Furthermore, losing or breaking up with someone close was another factor that accompanied feelings of sadness and depression.

#### b) Overthinking

Another highly common negative thinking style associated with the experience of loneliness was overthinking:

*“…it gets so sad and lonely in there, cause it’s you, it’s, my head is the most dangerous place cause then you get this this thought that just pops up out of nowhere like you are a bad person because you think this, in your own head you are like punching yourself for something that may not even be that significant but it’s your thoughts, you cannot really fight them, yeah, just get lost in your head is so lonely…”* [Female, 18, BAME, Barking and Dagenham]

Participants spoke about having two voices in their head that are constantly in conflict and create discomfort, i.e., “you should get up, you should do this, or you should do that. No, you need more sleep, go to bed,” or “let us go get lunch. No, you can wait.” Similarly, there was a mention of a voice that is constantly telling them “negative things” i.e., “you cannot do this,” “other people are better looking than you,” “you are not like other people.” Participants felt that no matter what they did, the negative voice could not be silenced. A number of them also said that not being able to express oneself and bottling up emotions inside led to excessive thinking. Furthermore, overthinking was connected to being lost in one’s thoughts, worry, and anxiety. Overall, the experience of overthinking was predominantly experienced when alone or in one’s bed at night; in these moments, participants constantly thought, questioned, and worried, particularly about what other people thought of them and whether they judged them. There was also mention of purposefully avoiding being alone to stop overthinking and also of overthinking at nighttime in bed disturbing sleep.

#### c) Fear

Fear was also a widespread emotion connected to the experience of loneliness, particularly the fear of being judged followed by the fear of being excluded, rejected, isolated, or left out. Young adults spoke about their fear of being judged by others based on how they look, speak, and act and that it might be easier to be alone because they did not have to worry about whether their behavior was right or wrong; they could be completely themselves without having to pretend. As a result of fear, they felt unable to talk about their feelings and issues and ended up keeping emotions inside and feeling upset. In many instances, the experience of fear was accompanied by worry, being bothered by one’s thoughts and doubting oneself and one’s worth. Both directly or indirectly, participants also expressed that they were under a great deal of pressure for acceptance regarding their physical appearance and having to follow expectations and trends; they feared being excluded, rejected, judged, isolated, or left out if they did not conform:

*“…like for me I might feel um stressed under the sense that um… “Am I not wearing the latest shoes?” Shoes are a big thing in today’s society, “are my shoes not clean?” and there’s just loads of pressures and expectations in today’s society that um if not, if not in line with, you can be what we feel to, seem as lonely, um so it’s the kind, like I said, it’s the kind of fear that if you are not looking a certain way, you are not having your muscles a certain way, you’ll be excluded, you’ll be isolated and then end up lonely and I think that’s what, um, the kind of feeling of fear and loneliness it stems from.”* [Male, 18, BAME, Hackney]

The data also showed that social media enhanced the sense of pressure participants experienced because platforms, such as Instagram, Snapchat, and Facebook emphasize appearance. These three social media platforms were specifically mentioned by a large number of participants, who saw them as detrimental.

#### d) Worry and Anxiety

The experience of loneliness was also accompanied by worry and anxiety, particularly for the female participants. When alone or outside in public by themselves, they worried or felt anxious about what other people thought of them, how they viewed them, whether they judged them or about if something bad happened to them:

*“I sit there and worry about nothing really, in that sense because it has not even happened yet, so why am I worrying, if it even happens at all, why am I worrying? I just worry about situations that probably will never happen, but yeah, I will think of every possibility. And it’s just… it upsets me as well because why am I the type of person that sits there and worrying like, I want to be not like a carefree, but like a carefree person where I can just sit there and not care about what others think and the labels on me or their opinions on me…”* [Female, 20, White, Barking and Dagenham]

A number of young adults also expressed being worried about money and what the future would hold for them:

*“…I’m thinking, am I gonna have money to get my own house and… am I gonna have money, like I wanna start driving, like um… do driving lessons and stuff, but I do not have the money to do that, and then I’ll think… um… and then you have got like other things that go along with a car, and it’s just a lot of money. I feel like a lot of my worries are money….”*[Female, 20, BAME, Barking and Dagenham]

#### e) Low Self-Worth

Low self-worth was another correlate of loneliness, particularly for young female participants. The data revealed that participants were under pressure to make an impact on others especially friends and family, achieve more and be viewed positively in the eyes of others. They constantly compared themselves with their peers thinking that others were more knowledgeable, popular, high-achieving, and successful. This comparison also triggered questioning or self-doubt and as such not “putting themselves out there” because they were worried about being judged. Collectively, these experiences reduced their confidence levels and led them to feeling low self-worth:

*“…like this weekend has really been like such a realization for me, when someone asked me like “what do you do?” and I just said “nothing” and I realized “wow, I’ve said that to so many people now”, just because I have nothing to say and it’s just this limbo part, me working in a cafe, like I have done a lot of creative work throughout the year, but it’s just like, right now, all I’m doing is working at the cafe but, um yeah, definitely makes you feel lesser and like you do not even have a reason to be alive…”* [Female, 20, White, Hackney]

#### f) Emptiness

A number of the participants also associated the experience of loneliness with the feeling of emptiness. A lack of meaning in life contributed to a sense of emptiness. In particular, they said that even if one had a family, friends, time to oneself, a career, status, material possessions, or a luxury life, it would not stop them from feeling empty or be enough to fill the void of loneliness they experienced:

*“So, um, box one, um, I drew a diagram. So, the stickman refers to an individual, and the circle is what’s around that person. So, in terms of loneliness, it does not matter you have seen around the people, even if they have family, friends, have a social life or having the time to themselves, a career, even those factors, they will not, they rarely fill that loneliness void…”* [Male, 23, BAME, Tower Hamlets]

The inner processes associated with the experience of loneliness were symbolized by one participant as a circle in one’s chest representing an empty feeling and one was trying to fill it up with many distractions:

*“So, this circle, when I thought about loneliness, I felt like this circle is in my chest, and it’s like this empty feeling in my chest and you are trying to fill it up, me, in particular, when I have this empty feeling, you know, you just, you are there, you are chilling and you try to fill up with many different kind of ways or distractions.”* [Male, 24, BAME, Newham]

### Theme 3: Coping Mechanisms

Just over half of the participants mentioned different coping mechanisms for loneliness either from their own experience or that of others. This was characterized by technological and non-technological outlets.

#### a) Technological Outlets

Technology was a predominant coping mechanism for loneliness among the participants, particularly listening to music, watching Netflix or TV, and going on one’s phone, the internet and social media as ways to distract oneself. The most common of these was listening to music, which was deemed an escape outlet from negative thoughts and loneliness experiences:

*“…when I’m alone, I would listen to music and like tidy up or something and… I find that sometimes fun for myself. “Cause I like‐ I enjoy listening to music, so I’ll entertain myself listening to music…”* [Female, 18, White, Hackney]

#### b) Non-technological Outlets

Coping mechanisms also included activities outside the realm of technology: seeing family and friends and engaging in non-verbal activities, such as reading, drawing, painting, writing, and dancing:

*“Well, drawing and painting is like an escape for me, or listening to music because even though there is some words that I could not, like express or through art… I do not have to use words to express how I feel, and I do not have to hurt anyone by expressing certain words, what I am thinking at the time, so music, painting and drawing is something that is so non-verbal that it would not hurt anyone…”* [Female, 20, White, Barking and Dagenham]

There were also mentions of coping mechanisms that participants saw as unhealthy such as using alcohol or drugs to deal with loneliness. Participants tended to attribute this coping mechanism to other people rather than to themselves. They also talked about it providing an escape from loneliness and one’s problems though making one feel worse in the long run.

Coping with one’s loneliness using alcohol or drugs was symbolized as a short-term fix or coverup:

*“Some people need to like drink alcohol so they can sleep. To silence their thoughts. It’s crazy, it’s the truth. People do drugs to silence their thoughts. But what you are doing‐ is it’s just your‐ it’s like‐ I’ll give you an analogy. It’s like you have damp on your walls. Rather than dealing with the problem outright, [you] paint over it. But after the coating comes off, the damp’s still there. The problem still remains.”* [Male, 24, BAME, Hackney]

### Theme 4: Positive Aloneness

#### a) Positive Aloneness in General

The final theme mentioned by more than half of the sample was positive aloneness, which is that being alone was not always a negative experience. In particular, participants drew a distinction between aloneness being a choice they were content with and loneliness as an uncomfortable experience:

*“I do not think being on your own is always a bad thing, I think it can be a good thing to be honest, but, um, maybe too much on your own can lead to feelings of loneliness. Yea, I mean I’m quite, I suppose in context as well, are you alone because you want to be alone or are you alone because you have to be alone?”* [Male, 24, BAME, Newham]

Despite the distinction made between aloneness and loneliness, some used the word “aloneness” and others “loneliness” when referring to either the negative or the positive experience of being on one’s own. While aloneness or loneliness was considered to be a positive experience at times, participants warned that too much alone time could be detrimental.

#### b) Time and Space for Reflection

The data also showed that the main reason aloneness was sometimes considered a positive experience was because it offered time and space for reflection. Specifically, many said that the experience of being alone or lonely allowed one to think, figure out things, do what one had to do, and ultimately provided a chance for growth and development:

*“…I did not really mingle or find the right people to mingle with [at university] so I found myself being lonely a lot of the times and it gets to the point whereby I got used to it, it’s just I became a loner if that’s the word in a way and I feel like being lonely as well kind of helped me to think about life in general, so I do not see loneliness as something that is a bad thing in a way yeah.”* [Female, 24, BAME, Barking and Dagenham]

Furthermore, there were mentions of being able to be completely oneself when one was lonely/alone without the fear of being judged by others, the pressure to fit in or having to put on a persona:

*“…it’s easier when there’s no correct, there’s no wrong, it’s just me, so I can be me. When I’m around people, I have to be what people want to see which as I said conveys the, conveys a person that’s not me…”* [Male, 24, White, Barking and Dagenham]

#### c) The Positive Experience of Social Media

In addition, social media played a positive role in many of the participants’ experience of positive aloneness. They enjoyed using social media because it enabled them to stay in touch with friends and family, watch funny videos and was a quick and convenient communication tool. Most commonly mentioned was connecting with friends:

*“Yeah, um so yeah, so I’ve got my iPad, my phone, I’ve got like lots of like different networking apps, so I feel socially connected to everything and everyone, really from like celebrities to n-, like outlets and like friends from across the country, to just kind of keep in touch with everyone, to keep in touch with my friends that I have now left, people are going off to university, it’s just, it’s just, using social media I can basically just keep connected to everyone and everything.”* [Female, 18, BAME, Newham]

Positive aloneness was symbolized as leaving one’s environment to find oneself:

*“…the first image is a very powerful image, “cause it’s not an image that I’ve come up with [in] my head, it is an image that I’ve seen once upon a time, but it resonates very well with me. But um… it’s‐ it’s the lone‐ it’s the lone wolf that… you know, strays away from the pack and decides to uh find oneself, you know. And then… when the time is right, maybe the paths could uh… criss-cross again, you know? That’s what the first image represents to me…”* [Male, 24, BAME, Hackney]

### Results of the Questionnaire-Based Analysis

#### The Revised UCLA Loneliness Scale

The mean total loneliness score on the Revised UCLA Loneliness Scale was 54.7 (*SD* = 7.08), which indicates a moderately high degree of loneliness.

#### Social Media Activity (General Social Media Usage)

The relationship between loneliness and social media activity was assessed using Pearson correlation. The test showed that the Revised UCLA Loneliness Scale and social media activity were not significantly correlated, *r*(46) = 0.01, *p* = 0.93.

#### Online Friendships

Similarly, to assess the relationship between online friendships and loneliness (UCLA Loneliness Scale), a Pearson correlation test showed that the two were not significantly correlated, *r*(46) = ‒0.04, *p* = 0.80.

**Figure 2:**
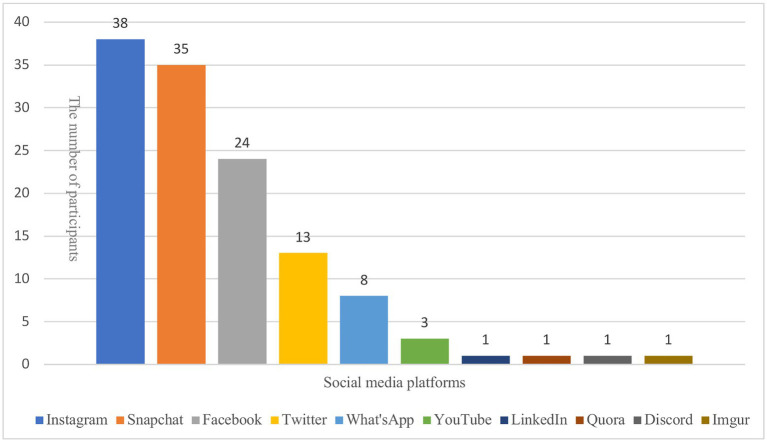
The popularity of social media types based on the number of participants using them.

In addition, participants were asked what social media channels they use and to list their top three based on frequency of use. Figure 2 shows the number of participants who used the different types of social media channels.

## Discussion

This study aimed to address what the experience of loneliness is in young adults from London’s most deprived areas, how they cope and whether the social media impact their loneliness. First and foremost, the quantitative data indicated that the sample was indeed lonely, in line with findings that young adults, particularly those from deprived areas with lower SES are a lonely demographic in the United Kingdom. They also corroborate findings from other studies, indicating that young adults are lonely in contemporary times (Cigna, 2018, 2020; DiJulio et al., 2018; Office for National Statistics, 2018a; YouGov, 2019).

The experience of loneliness was associated with being isolated or excluded both physically and emotionally; this could occur even when surrounded by people. This indicates that young adults feel a lack of both a social network and intimate or close relationships. This is consistent with multidimensional theory of loneliness of Weiss (1973), which proposes both social and emotional types of loneliness.

The present study did not find a significant relationship between loneliness and social media use or online friendships. Both the quantitative and qualitative aspects of the study suggest that loneliness in young adults may be less dependent on the extent of social media usage or number of online friends but more on how the outlets are used. This sheds further light on the equivocal findings in the literature concerning social media’s impact on loneliness and corroborates a previous meta-analysis (e.g., Liu et al., 2019). Finally, the most frequent social media channels that the young adults used were Instagram, Snapchat, and Facebook, respectively. Even though Instagram is ranked the worst social media platform for mental health (Cramer, 2018), participants considered it their top choice in line with other research (e.g., Auxier and Anderson, 2021).

Social media played a role in magnifying the sense of isolation, particularly when participants saw images of their friends at occasions to which they had not been included. Their experience of loneliness was accompanied by a set of interrelated negative feelings and thoughts such as sadness and overthinking. In order to cope with these, young adults either turned to technology or relied on non-technological means.

Finally, loneliness was not always considered a negative experience by young adults because it gave them time and space for reflection and ultimately the opportunity for growth. Moreover, while social media exacerbated loneliness in the main, it could also link young adults to others and bring laughter and entertainment. In the present study, however, the focus tended to be more negative than positive regarding social media’s impact on loneliness.

Qualitative responses were compared across gender and ethnicity. Overall, there was strong homogeneity across the sample; however, young women were more likely to engage in negative emotions and thoughts than young men were, particularly when it came to worry and anxiety, and having low self-worth. This is consistent with existing literature that women are more likely to experience negative emotions (i.e., see Brody and Hall, 2008; McLean and Anderson, 2009; Else-Quest et al., 2012; Caballo et al., 2014). There were also proportionally more white young adults who spoke about coping with the experience of loneliness than BAME counterparts. This included proportionally more talk of both technological and non-technological coping mechanisms. Interestingly, despite the women’s greater preponderance to negative emotions regarding loneliness, they were more likely to talk about the positive experience of aloneness than their male counterparts. Perhaps this reflects a greater focus on emotion – negative and positive – among women. Future researchers might examine these potential trends more closely.

### The Experience of Loneliness and Coping

The present study’s finding corroborated the evolutionary theory of loneliness in two ways: first regarding the association between the experience of loneliness and increased hypervigilance for social threats. What this study added is the application of the theory to young adults’ experience of loneliness in relation to the fear of being judged both offline and online. This study showed that loneliness in young adults is associated with the fear of being judged or rejected by others; this experience signals perceived threat to sense of self and causes intentional isolation to protect the self. Social media magnified young adults’ hypervigilance to social cues and perceived threat to their identities since they emphasized how one is portrayed to others. Second, the present findings regarding coping with loneliness support evolutionary theory in terms of seeking social bonds when one feels lonely as loneliness is a signal that social connections are absent and motivates reconnection with others. Many young adults in the present study were motivated to turn to family and friends both offline and online as a way to cope with their experience of loneliness.

The present study was also consistent with the ONS’ findings in terms of the link between loneliness and mental health challenges such as depression and anxiety. What the current study added to previous research was that young adults can feel lonely even when surrounded by family and friends. Their experience of loneliness is accompanied by negative emotions and thoughts such as fear and overthinking. Moreover, they use technological and non-technological outlets in order to cope with their loneliness. Finally, social media play a role in amplifying the fear of judgment and exclusion.

The finding in relation to the positive experiences of being alone was also in line with previous research (e.g., Storr, 1988; Larson, 1997; Galanaki, 2013; Goossens, 2014). This study further adds that being alone for young adults is associated with being themselves without the fear of being judged or the pressure to be accepted by others.

Furthermore, this study’s findings corroborated Woodward and Frank’s (1988) and Vasileiou et al. (2019) studies in relation to seeking support from family and friends, engaging in pleasurable and constructive activities, and going on the internet and social media and substance use. In line with Seepersad (2004), the current findings showed that online coping with loneliness reflects, and is an extension of, offline coping such that young adults sought out family and friends as well as to entertain themselves both offline and online in order to deal with their loneliness. Not corroborated was coping by way of owning pets, physical exercise, and using positive affirmations. This is very important because young, employed people, of lower SES, and living in deprived areas may not have the financial resources or time to care for pets or engage in physical exercise. This was supported by the mentions of money problems and the pressure of working. Hence, this study served to highlight how the lack of resources such as money and time can deprive people of coping outlets.

### Social Media

Consistent with previous research concerning the impact of social media on wellbeing (Liu et al., 2019), the current findings indicated that passive consumption of social media such as browsing and observing others but not interacting with them was associated with the experience of loneliness. Conversely, the current study also supported Deters and Mehl (2013) finding in relation to the positive impact of social media on loneliness, when connecting with friends. The current study provides a qualitative perspective on the equivocal quantitative findings. It shows that the way in which one engages with social media determines whether it is detrimental or beneficial to one’s wellbeing: active engagement is experienced as beneficial while passive engagement is not. Indeed, the current study pinpoints social media as a magnifier of fears of judgment and the pressure young adults experience given the social media’s emphasis on popularity and promoting one’s profile.

### Theoretical Implications

In light of research showing young adults, particularly from deprived areas, to be the loneliest demographic in the United Kingdom, the current study was the first to explore the experience of loneliness within this group. Since the present study used a novel free association method with antecedents in the psychoanalytic tradition, it tapped thoughts, feelings, and non-conscious processes regarding young adults’ experience with minimal researcher-led content. Hence, the study provided a more naturalistic way of gaining an understanding of the sample’s experience of loneliness and revealed a large range of symbols that participants used to explain their experience– *the caged lion* trapped in a perspective that is different to others’ and acting as a form of entertainment for these others; *an empty circle in one’s chest* signifying a void; *painting over the damp on the wall* rather than fixing the issue; and a *lone wolf* who strays from others to find itself. Thus, phenomenological and free associative strands worked together to reveal deep-laid layers of thinking and feelings.

Nevertheless, there was a limitation to the study. It cannot reveal whether the themes found are specific to young adults in deprived areas as it did not compare the responses to those of young adults in non-deprived areas. Hence, a study examining a matched sample in non-deprived areas would be instructive.

### Practical Implications

In terms of practical relevance, based on the findings, Personal, Social and Health Education (PSHE) lessons, which form part of the United Kingdom’s national school curriculum, should incorporate the potential impact of social media on loneliness and wellbeing; children and young adults should be made aware that passive use tends to have negative consequences for their sense of loneliness while active use can have positive consequences. Educational institutions could also provide a non-judgmental space and offer cognitive behavioral therapy as it has been meta-analytically shown to be an effective intervention for young adults struggling with negative emotions and thoughts, such as anxiety and depression (Pennant et al., 2015). Making it known that times of aloneness can be positive in that they allow for reflection and potential growth should further be included in loneliness reduction strategies to help young adults use their solitary time more fruitfully.

Similarly, charities that work with children and young adults could consider disseminating videos to highlight how to engage with social media in a way that fosters wellbeing. Online videos have been shown to be of particular interest to younger people (Rideout and Robb, 2019). Social media platforms should focus on offering features that enhance active communication. This is consistent with the stimulation hypothesis, which posits that social media can stimulate the quality of existing relationships by extending them to online settings and create opportunities to form new friendships, thereby reducing loneliness (e.g., Shaw and Gant, 2002; Valkenburg and Peter, 2007; Skues et al., 2012; Lemieux et al., 2013).

Given that some young adults experience a sense of emptiness at times, educational institutions could further deliver workshops to help them find out what gives them meaning in life. Parents, schools, work environments, and the government should also consider providing opportunities for young adults to engage in meaningful activities such as volunteering and helping others in order to reduce feelings of emptiness. Volunteering enhances adults’ sense that their lives have meaning (Greenfield and Marks, 2004).

As for coping mechanisms, the data from the current study indicated that young adults use technological platforms to distract themselves or escape from feeling lonely. While these coping strategies may be effective in the short-term, they do not help to overcome loneliness in the longer term. It is more sustaining to find activities that offer a sense of purpose. Activities, such as reading, drawing, or dancing can create the experience of “flow” and diminish self-conscious rumination, giving enjoyment, meaning, and growth (Csikszentmihalyi, 1990, 2013). Seeing friends and family is another effective coping mechanism, which should be encouraged among young adults because there is considerable, consistent evidence that social support is a major contributor to wellbeing (Diener and Seligman, 2002; Jenkins et al., 2008). This is also consistent with social determination theory, which posits that relating to others is a basic psychological need (Ryan and Deci, 2000). Interventions to help young adults cope with their experience of loneliness should focus on activities that foster meaning, growth, and social connectedness.

Loneliness has become a major contemporary health concern and young adults are currently the loneliest of all age-groups within the United Kingdom and beyond. The present study has provided the first systematic, in-depth analysis of the subjective experience of loneliness among those who fit within the loneliest demographic. Young adults from London’s most deprived communities can feel isolated even in the company of family and friends; they sometimes engage in a set of interrelated emotions and thoughts that engender discomfort. Listening to music often provides a sense of distraction but is unlikely to be effective in tackling the experience of loneliness in the long run. However, seeing friends and family and engaging in meaningful activities may do so. Researchers are invited to longitudinally examine the effects of the different coping mechanisms in order to better inform interventions targeted at diminishing loneliness.

## Data Availability Statement

Requests for raw data supporting the conclusions will be considered.

## Ethics Statement

The studies involving human participants were reviewed and approved by UCL Research Ethics Committee (CEHP/2013/500). The patients/participants provided their written informed consent to participate in this study.

## Author Contributions

SF and HJ were involved in the creation and development of the study. SF collected the data. SF and HJ analyzed the data and wrote the manuscript. Both authors approved the final version to be published and agree to be accountable for the content of the work.

### Conflict of Interest

The authors declare that the research was conducted in the absence of any commercial or financial relationships that could be construed as a potential conflict of interest.

## Footnotes

^1^There were two parts to the study and the focus in this study is on this first part.

^2^The calculation was made on the basis of whether participants talked about a particular theme or not. When 30 percent more of a group being compared to another (e.g., males versus females) mentioned a theme, this was regarded as a noteworthy difference.
